# Survival prediction models: an introduction to discrete-time modeling

**DOI:** 10.1186/s12874-022-01679-6

**Published:** 2022-07-26

**Authors:** Krithika Suresh, Cameron Severn, Debashis Ghosh

**Affiliations:** 1 Department of Biostatistics and Informatics, Colorado School of Public Health, Aurora, USA; 2 Child Health Biostatistics Core Department of Pediatrics, Section of Endocrinology, School of Medicine, University of Colorado Anschutz Medical Campus, Aurora, USA

**Keywords:** Cox proportional hazards, Machine learning, Random survival forest, Time-to-event

## Abstract

**Background:**

Prediction models for time-to-event outcomes are commonly used in biomedical research to obtain subject-specific probabilities that aid in making important clinical care decisions. There are several regression and machine learning methods for building these models that have been designed or modified to account for the censoring that occurs in time-to-event data. Discrete-time survival models, which have often been overlooked in the literature, provide an alternative approach for predictive modeling in the presence of censoring with limited loss in predictive accuracy. These models can take advantage of the range of nonparametric machine learning classification algorithms and their available software to predict survival outcomes.

**Methods:**

Discrete-time survival models are applied to a person-period data set to predict the hazard of experiencing the failure event in pre-specified time intervals. This framework allows for any binary classification method to be applied to predict these conditional survival probabilities. Using time-dependent performance metrics that account for censoring, we compare the predictions from parametric and machine learning classification approaches applied within the discrete time-to-event framework to those from continuous-time survival prediction models. We outline the process for training and validating discrete-time prediction models, and demonstrate its application using the open-source R statistical programming environment.

**Results:**

Using publicly available data sets, we show that some discrete-time prediction models achieve better prediction performance than the continuous-time Cox proportional hazards model. Random survival forests, a machine learning algorithm adapted to survival data, also had improved performance compared to the Cox model, but was sometimes outperformed by the discrete-time approaches. In comparing the binary classification methods in the discrete time-to-event framework, the relative performance of the different methods varied depending on the data set.

**Conclusions:**

We present a guide for developing survival prediction models using discrete-time methods and assessing their predictive performance with the aim of encouraging their use in medical research settings. These methods can be applied to data sets that have continuous time-to-event outcomes and multiple clinical predictors. They can also be extended to accommodate new binary classification algorithms as they become available. We provide R code for fitting discrete-time survival prediction models in a github repository.

**Supplementary Information:**

The online version contains supplementary material available at (10.1186/s12874-022-01679-6).

## Background

Survival prediction models are often used at some baseline time, such as time of diagnosis or treatment, to answer questions such as “What is the probability that this patient will be alive in 5 years, given their baseline covariate information?” This predicted probability can then be used by clinicians to make important decisions regarding patient care, such as increasing monitoring frequency or implementing particular therapies. Survival prediction models are built using time-to-event data, which includes the time that an individual is observed for the event of interest and whether they experience the event at the end of that follow-up time. For some individuals, we do not observe the event of interest during their follow-up so we have incomplete information about these individuals. That is, we do not know when the event occurs but we know that it happens sometime after their observation period. These are considered to be *censored* observations, and to exclude or ignore this information when analyzing the data can lead to biased and inefficient predictions [1]. Thus, survival prediction models differ from traditional prediction models for continuous or binary outcomes by appropriately accommodating censoring that is present in time-to-event data.

There is a vast class of survival prediction models, ranging from those built using traditional regression methods to more recently developed machine learning algorithms [2]. The traditional approaches treat time-to-event as a continuous outcome and make parametric or semi-parametric assumptions about the distribution of the survival times. Parametric survival models assume a particular distribution for the survival times (e.g., Weibull, exponential), which is often based on prior knowledge of the clinical or scientific context. These models can be more efficient and accurate when the survival times follow the assumed parametric distribution but can lead to biased estimates when misspecified [3]. The semi-parametric Cox proportional hazards model leaves the distribution of the survival times to be estimated using non-parametric methods, such as the Breslow estimator, but incorporates covariate effects so that individuals with different sets of predictors can have different predicted survival curves [4]. The standard Cox model relies on a proportional hazards assumption for the survival times that when violated can lead to inaccurate predictions. This violation can be common in practice when there are time-varying covariate effects and unobserved heterogeneity [5].

An increasingly common approach to building survival prediction models are machine learning (ML) algorithms that use mathematical procedures to model the relationships between covariates. Common ML algorithms for survival have been extended from those developed for classification problems or traditional survival models and include penalized or boosted Cox regression [6, 7], random survival trees and forests [8–11], support vector methods [12, 13], and artificial neural networks [14–17]. The advantages of ML approaches are that they are algorithms designed for achieving optimal predictive performance, they do not require specification of covariate relationships, and they are able to capture complex and nonlinear relationships in the data. As well, unlike parametric and semi-parametric survival models, they do not make distributional or proportional hazards assumptions about the survival times. The disadvantages include lack of interpretability, computational intensity, and overfitting that results in poor external validation. In situations of high-dimensionality or complex covariate relationships, ML methods have been shown to have superior predictive performance compared to traditional regression-based approaches. However, in application to time-to-event data, machine learning algorithms can have mixed performance for predicting survival outcomes in comparison with Cox regression models [1, 18, 19]. Thus, the extent to which these machine learning approaches are necessary or useful may depend on the particular application and data set.

The survival prediction models that we have described thus far are built using methods or algorithms for continuous time-to-event outcomes, while accommodating censored observations. These include ML algorithms originally built for classification or continuous outcomes that have been adapted to accommodate censoring. Unlike classification models that have been implemented in unified R packages, such as ‘caret’ and ‘h2o’, survival prediction models are usually hosted in separate packages (e.g., ‘survival’, ‘fastcox’, ‘randomForestSRC’ in R) and often in different software (e.g., ‘PySurvival’ in Python) [20–25]. Thus, to take advantage of the predictive power of existing ML classification algorithms and their available and unified software, we consider a class of discrete-time survival prediction models that are built using classification prediction models but are able to take into account censoring.

In a discrete-time survival framework [26, 27], we can transform continuous survival time data into a discrete-time format using a person-period data set. Each individual’s follow-up time is split into a set of pre-specified, common time intervals, and their survival status is recorded for each of the intervals during which they are at risk (i.e., have not yet experienced the event of interest). The general idea of prediction using this discrete-time framework is to build models that predict the probability of surviving each of these discrete-time intervals, which when treated as a binary outcome of experiencing an event within the interval can be framed as a series of binary classification problems. This discrete-time formulation of survival data is general such that any binary classification method can be applied, allowing for a wide range of models to be considered.

Traditional approaches fit a logistic regression model to this data [4, 27, 28]. However, these models require specification of covariate relationships with outcomes, and thus knowledge of covariate interactions and behavior. Instead, we demonstrate the use of ML classification methods, such as random forest or neural networks, to predict the conditional survival probabilities for each of the intervals. Using these predictions, we can then compute our quantity of interest, which is an individual’s probability of surviving beyond a certain time. We present the process for building and testing a discrete-time survival model using this approach and demonstrate it using two publicly available data sets. With these data, we compare the predictive performance of various classification approaches applied in the discrete-time framework and common survival prediction models, such as Cox regression and random survival forests.

The use of a discrete-time survival framework for prediction is not recent. ML classification algorithms applied to a discrete-time framework has been demonstrated using tree-based methods [29, 30] and neural networks [31–34]. However, the use of discrete-time survival for prediction is uncommon in clinical applications, with models built using continuous-time methods such as Cox proportional hazards (PH) or random survival forests being more popular. With the increasing interest in exploration of ML algorithms for predicting survival outcomes, a discrete time-to-event method using machine learning provides a potentially powerful and easily implementable alternative approach. This manuscript contributes to the existing literature by presenting a guide for the development and assessment of discrete-time survival models built using ML classification algorithms for survival prediction, and providing code for its implementation in R software. We demonstrate that there are settings in which these models can achieve superior predictive performance compared to continuous-time models, and thus should be considered as candidates in the development of survival prediction models.

### Primary aim

This paper provides a demonstration for using discrete-time modeling for obtaining survival predictions in the context of time-to-event outcomes. We describe the methodology and application of binary classification models to predict conditional survival probabilities. We present the process for building and testing discrete-time prediction models. We additionally make available R code for implementing this process and for comparing the predictive performance to common continuous-time survival models. The primary goal is to provide applied statisticians with resources for training and evaluating discrete-time prediction models to encourage their use in medical research for personalized decision-making.

### Structure of this paper

This manuscript is presented as a tutorial for building survival prediction models using discrete-time modeling. We assume the reader already has basic knowledge of survival analysis and predictive modeling. First, an introduction is given to prediction with machine learning algorithms and prediction models built to accommodate right-censored time-to-event data. Within this class of models, we present parametric survival models, the commonly used Cox proportional hazards model, and machine learning survival algorithms, such as the random survival forest. Second, we describe discrete-time survival modeling using binary classification models and how it can be used for prediction. Third, details are provided on two commonly used performance metrics for assessing survival prediction models, Area under the receiver operating characteristic (ROC) curve (AUC) and Brier score (BS). Fourth, we present a guide for building and validating discrete-time prediction models. Using publicly available data sets, we demonstrate our approach using code written for the R programming language [35]. Finally, we end with a discussion of the advantages of this approach and the opportunities it provides for future research.

## Machine learning and continuous-Time prediction models

### Machine learning algorithms

Machine learning algorithms capture the relationships between covariates by solving mathematical procedures using numerical computation. The primary goal of building a ML algorithm is prediction. These algorithms use iterative optimization techniques to flexibly model non-linear and complex relationships between covariates to minimize prediction error. This can make it difficult to then extract and interpret covariate effects and interactions from them. This is in contrast to the traditional statistical methods that are developed for inference and interpretability, but can be used for prediction as well.

We consider the class of supervised ML algorithms, where we are interested in building a model using a set of covariates. We apply this trained model to a new subject to obtain predictions. Thus, instead of interpretation, our goal is to build a model with superior predictive performance. We explore the application of machine learning algorithms in two ways. First, we consider survival ML algorithms that have been adapted to accommodate right-censored data, such as random survival forests. Second, we consider classification algorithms that predict a binary outcome that can be applied in a discrete-time framework. The purpose of exploring machine learning alternatives to traditional statistical methods in predicting survival is to present an approach that is designed to achieve superior predictive performance and can therefore be reliably used in clinical decision-making. In Table 1, we briefly describe some commonly used ML algorithms, but readers that are interested in a detailed introduction to machine learning are referred to [36–40].

**Table 1:** Machine learning methods

Method	Description
Random forest	Are an ensemble of tree-based learners that are built using bootstrap samples of the training data and average the predictions from the individuals trees. In constructing the trees, a random subset of features is selected for evaluating the split criterion at each node. This leads to de-correlated individual trees that can improve predictive performance.
Boosting	Are an ensemble of base learners that are constructed sequentially and are progressively reweighted to increase emphasis on observations with wrong predictions and high errors. Thus, the subsequent learners are more likely to correctly classify these misclassified observations.
Support vector machines	Uses a kernel function to map input features into high-dimensional feature spaces where classification (survival) can be described by a hyperplane.
Penalized regression	Provides a mathematical solution to applying regression methods to correlated features by using an *ℓ*_2_ penalty term (ridge). Additionally, can encourage sparsity by using an *ℓ*_1_ penalty (LASSO) to avoid overfitting and perform variable selection. A weighted combination of *ℓ*_1_ and *ℓ*_2_ penalties can be used to do both (elastic net).
Artificial neural networks	Are comprised of node layers starting with input layer representing the data features, that feeds into one or more hidden layers, and ends with an output layer that presents the final prediction.

### Continuous-Time survival models

In the survival setting, the observed data is given by $\mathcal {D}_{n}=\{\tilde {T}_{i},\delta _{i},X_{i};i=1,...,n\}$ where $\tilde {T}_{i}=\min (T_{i},C_{i})$ is the observed event time for the *i*th subject (*i*=1,...,*n*), with *T*_*i*_ denoting the true event time, *C*_*i*_ the censoring time, *δ*_*i*_=*I*(*T*_*i*_≤*C*_*i*_) the event indicator, and *X*_*i*_≡(*X*_*i*1_,...,*X*_*iJ*_)^′^ the observed baseline covariate vector. We consider the common situation of a right-censored survival outcome, where if an individual’s observed survival time is censored we know only that they experienced the event beyond that time. We also assume independent or noninformative censoring conditional on covariates, such that *T*_*i*_ and *C*_*i*_ are independent random variables given *X*_*i*_.

A key quantity associated with time-to-event outcomes is the survival function, *S*(*t*)=Pr(*T*>*t*), which is a function of time, *t*, and is the probability of surviving beyond that time. This function describes a patient’s survival curve during follow-up. For a patient *k* with predictors *X*_*k*_, the quantity of interest is then denoted by *π*_*k*_(*t*)=Pr(*T*>*t*|*X*_*k*_). Another important characteristic of survival models is the hazard function, denoted *h*(*t*), which represents the instantaneous risk of experiencing the event at time *t*, given that the individual has not experienced the event up to that time. The hazard function is defined as *h*(*t*)=*f*(*t*)/*S*(*t*), where *f*(*t*) is the density function for the observed event time. Additionally, we define the cumulative baseline hazard $H(t)=\int _{0}^{t} h(s) ds$ and can write the survival function as *S*(*t*)= exp{−*H*(*t*)}. Survival prediction models commonly predict a survival function from which we can directly obtain *π*_*k*_(*t*), or a prognostic index (e.g., the linear predictor) that can be transformed to obtain the survival distribution.

In this section, we begin by describing some common regression and ML approaches for building survival prediction models using parametric, semi-parametric, or non-parametric methods.

#### Parametric survival models

Parametric survival models assume that the survival times follow a particular distribution that can be specified as a function with finite-dimensional parameters. One approach is to specify a particular distributional family for the survival time and then allow the parameters of that distribution to depend on covariates. Thus, the functional form for the model is completely specified while parameter values are unknown. For example, if we assume that the survival times were exponentially distributed, i.e., *f*(*t*)=*λ* exp{−*λt*} and *h*(*t*)=*λ*, we can let the rate parameter depend on covariates using *λ*= exp{*βX*}. Other parametric families that are commonly considered for modeling survival times are Weibull, exponential, log-logistic, log-normal, and the generalized gamma [3].

Many parametric survival models (e.g., Weibull, exponential) are also accelerated failure time (AFT) models. That is, covariates have a multiplicative (i.e., proportional) effect on the survival time [3], and we assume that the relationship between the log of the survival time and the covariates is linear, i.e., log(*T*)=*βX*+*σW*, where *σ* is a scale parameter and *W* is an error term. Thus, the main difference between this class of models and linear regression methods applied directly to the event times is the inclusion of censored observations in the estimation procedure. The type of AFT model is determined by the distribution assumed for *W* (e.g., an extreme value distribution leads to a Weibull model for *T*).

Alternatively, we can model survival data using a proportional hazards model. In contrast to AFT models, in PH models the covariate effects are multiplicative with respect to the hazard, rather than the survival. Thus, *h*(*t*)=*h*_0_(*t*) exp{*βX*}, where *h*_0_(*t*) is the baseline hazard (i.e., the hazard when the covariates are at their reference level). The specific proportional hazards model is defined by the parametric form selected for the baseline hazard. If we assume the baseline hazard is exponential, i.e., *h*_0_(*t*)=*λ*, then the hazard is *h*(*t*)=*λ* exp{*βX*}, which is actually still an exponential model since the hazard is constant. Similarly the Weibull baseline hazard results in a Weibull model. However, that is not the case for all distributions (e.g., a log-logistic baseline hazard does not result in a log-logistic PH model).

Estimation for parametric survival models can be conducted using maximum likelihood estimation, which is implemented using the survreg function in the R software package ‘survival’ [22]. An advantage of these models is that in addition to predicting a patient’s survival probability, we can also predict their survival time, which is not directly possible with the other survival models we will cover. As well, these models can be more efficient when the survival times follow a particular distribution. However, the disadvantage is that if the distribution is not correctly specified then results can be biased [3], which can affect predictive performance.

#### Cox proportional hazards model

We can instead consider the popular Cox PH model, which is a semi-parametric model where the distribution of the outcome is unknown. These models are not fully parametric since, while we specify regression parameters for the covariate effects, the baseline hazard (or baseline survival) is not specified. Thus, the Cox model is a generalization of the parametric proportional hazards model. The advantage of the Cox model is that it does not rely on distributional assumptions for the survival times.

In Cox PH models, the hazard function is modeled as *h*(*t*)=*h*_0_(*t*) exp{*βX*}, where *β* is a vector of regression coefficients and *h*_0_(*t*) is a nonparametric baseline hazard. The predicted survival probability of interest is then obtained from this model as $\pi _{k}^{Cox}(t)=\exp \{-H_{0}(t)e^{\beta X_{k}}\}$, where $H_{0}(t)=\int _{0}^{t} h_{0}(s) ds$ is the cumulative baseline hazard function. The *β* coefficients are estimated using maximum likelihood estimation of a partial log likelihood in which the baseline hazard *h*_0_(*t*) is left unspecified and treated as a nuisance function. The primary purpose of Cox regression is to identify association between risk factors and outcomes, and thus a second step must be taken to estimate the baseline hazard function required for prediction. The cumulative baseline hazard function *H*_0_(*t*) is estimated using a non-parametric method, most commonly the Breslow estimator.

To estimate both the regression coefficients and the cumulative baseline hazard, we use the observed survival times *T* to identify risk sets, which are comprised of subjects still at risk of the event, at unique event times in the data set. Thus, the estimation depends on the ordering of survival times rather than their specific values. If there are ties in the event times (e.g., if failure times are only reported to the nearest day), then the survival times cannot be uniquely sorted and an approximation of the partial likelihood function is required for estimation [3]. If there are many ties, then it may make more sense to treat the failure time distribution as discrete and use a discrete-time framework. For more details about the estimation of Cox PH models, see [3, 4]. Estimates of *β* and *H*_0_(*t*) are then plugged into $\pi _{k}^{Cox}(t)$ to predict the individual’s probability of surviving beyond time *t*. Estimation for a Cox PH model is implemented by the coxph function in the R package ‘survival’, and predictions from these models can be obtained using the predictCox function in the R package ‘riskRegression’ [41].

#### Machine learning survival algorithms

Several ML algorithms have been developed to handle censored time-to-event data. With these methods we can build survival prediction models for high-dimensional, complex data that can have greater accuracy than traditional regression methods. Existing ML algorithms for survival include penalized Cox regression, boosted Cox regression, survival trees and random survival forests, support vector regression, and neural networks. These methods are extensions or adaptations to the ML algorithms presented in Table 1 and are described in further detail in [2].

ML methods that are based on penalized or boosted Cox regression suffer from the same issues as the Cox PH model, in that they need a second step in software implementation to estimate the baseline hazard function for predicting survival probabilities and still rely on the proportional hazards assumption. Support vector regression has been extended for survival analyses using two approaches: (i) ranking, which predicts the risk ranks between subjects, and (ii) regression, which predicts a subject’s survival time, both of which are considered in [13]. Neither of these support vector machine (SVM) approaches produces predictions that are related to the survival or cumulative hazard function, and thus cannot be used to obtain a predicted survival probability. Neural networks for survival have expanded on the Cox PH model [14, 42, 43], but again only output a prognostic index and not the survival probability, thus requiring additional estimation of the baseline hazard using the Breslow estimator. In this tutorial, to present a comparison to a ML method extended to accommodate censoring we focus on the commonly used approach for survival prediction, the random survival forest.

#### Random survival forests

Random survival forests are an ensemble method based on the bagging of survival trees [8]. A survival tree is a decision tree that is built by recursively splitting tree nodes based on a particular feature. Each split is made using a dissimilarity measure that computes the survival difference between the two new nodes and selects the best split as being the one that maximizes this survival difference. This dissimilarity measure is often selected to be the test statistic of the log rank test [8]. With random survival forests, we take *b*=1,…,*B* bootstrap samples from the original data set. For each bootstrap sample *b*, we grow a survival tree, where for each node we consider a set of randomly selected *p* candidate predictors rather than the full set of predictors, and split the node on the predictor that maximizes the dissimilarity measure. We grow the survival tree until each terminal node has no fewer than *d*_0_ unique deaths. In each terminal node, we compute the conditional cumulative hazard function using the Nelson-Aalen estimator, a non-parametric estimator of the survival function, using the subjects that are in the bootstrap sample *b* whose predictors place them in that terminal node.

To obtain a prediction for an individual *k* with predictors *X*_*k*_, for the survival tree created using bootstrap sample *b*=1,…,*B* we obtain the conditional cumulative hazard function of the terminal node that individual *k* belongs to based on their predictors, denoted *H*^(*b*)^(*t*|*X*_*k*_). We then compute their ensemble cumulative hazard function as $H_{k}(t)=1/B\sum _{b=1}^{B}H^{(b)}(t|X_{k})$. The predicted survival probability of interest is then given by $\pi ^{RSF}_{k}(t)=\exp \{-H_{k}(t)\}$. A random survival forest can be fit in R software using the ‘randomForestSRC’ package [24], which contains a predict function to compute predicted probabilities. This function allows the user to specify the rule used to determine the node split, and by default uses log-rank splitting [44]. It also requires the specification of hyperparameters, such as terminal node size (*d*_0_) and the number of variables to randomly select for each split (*p*), which can be optimally identified using hyperparameter tuning.

## Methods

### Discrete-time survival models

The methods we have discussed so far are only applicable to continuous survival times. In the discrete-time framework, we assume that the available data is the same but we define the hazard function and the link between the hazard and survival functions differently. We divide the continuous survival time into a sequence of *J* contiguous time intervals (*t*_0_,*t*_1_],(*t*_1_,*t*_2_],…,(*t*_*J*−1_,*t*_*J*_], where *t*_0_=0. Within this framework the hazard, or instantaneous risk of the event, in a particular interval is the probability of an individual experiencing the event during that interval given that they have survived up to the start of that interval. So, in discrete time, the hazard is a conditional probability rather than a rate, and as such its value lies between zero and one. Thus, for an individual with baseline covariates *X*_*i*_, the hazard in interval *A*_*j*_=(*t*_*j*−1_,*t*_*j*_] can be expressed as the conditional probability

$$ \begin{aligned} \lambda_{ij}(X_{i})&=\text{Pr}(T_{i}\in A_{j}|T_{i}>t_{j-1},X_{i})\\ &= \text{Pr}(t_{j-1}<T_{i}\leq t_{j}|T_{i}>t_{j-1},X_{i}) \end{aligned} $$

and the discrete probability function is given by

$$f_{ij}=\text{Pr}(T_{i}\in A_{j}|X_{i}) = S(t_{j-1}|X_{i})- S(t_{j}|X_{i}) $$

The probability of surviving beyond a particular time *t* can be obtained as the product of the conditional survival probabilities for all the time intervals up to and including (*t*_*j*−1_,*t*_*j*_], such that *t*_*j*_≤*t*. This is analogous to specifying the survival function in continuous time as the integrated hazard over all previous times. Thus, the survival probability in discrete time is given by

1 $$  S_{i}(t|X_{i}) = \text{Pr}(T_{i}>t|X_{i})=\prod_{j:t_{j}\leq t}(1-\lambda_{ij}(X_{i}))  $$

Note that the relationship *λ*_*ij*_(*X*_*i*_)=*f*_*ij*_/*S*_*i*_(*t*_*j*−1_|*X*_*i*_) still holds under these definitions. To construct the likelihood, subject *i* contributes the product of the conditional survival probabilities for the time intervals in which they are observed but do not experience the event. Individuals that are observed to have a failure (i.e., *δ*_*i*_=1) additionally contribute the conditional failure probability in the interval $A_{j_{i}}=(t_{j_{i}-1},t_{j_{i}}]$ in which they experience the event of interest. We use *j*_*i*_ to denote the last interval during which we have information about subject *i*, such that $\phantom {\dot {i}\!}T_{i}\in A_{j_{i}}$. Subject *i* does not contribute any information to the likelihood for intervals beyond $\phantom {\dot {i}\!}A_{j_{i}}$. Here, we only consider right-censoring, so the likelihood is given by

$$\begin{array}{*{20}l} {}L&=\!\prod_{i=1}^{n} \left[\text{Pr}(T_{i}=t_{j_{i}})\right]^{\delta_{i}}\left[\text{Pr}(T_{i}>t_{j_{i}})\right]^{1-\delta_{i}}\\ {}&=\!\prod_{i=1}^{n}\left[\!\lambda_{ij_{i}}(X_{i})\prod_{j=1}^{j_{i}-1}(1-\lambda_{ij}(X_{i}))\!\right]^{\delta_{i}}\left[\prod_{j=1}^{j_{i}}(1-\lambda_{ij}(X_{i}))\right]^{1-\delta_{i}} \end{array} $$

where $t_{j_{i}}$ indicates that subject *i* has a survival time in interval $\phantom {\dot {i}\!}(t_{j_{i}-1}, t_{j_{i}}]$. We can introduce an event history indicator $\phantom {\dot {i}\!}d_{ij}=I(T_{i}\in A_{j_{i}})=I(t_{j-1}<T_{i}\leq t_{j})$, which for censored subjects is given by $\phantom {\dot {i}\!}(d_{i1},\hdots,d_{ij_{i}})=(0,\hdots, 0)$ and for subjects that experience the event is $\phantom {\dot {i}\!}(d_{i1},\hdots,d_{ij_{i}})=(0,\hdots, 0, 1)$. The likelihood can then be written as

2 $$  L=\prod_{i=1}^{n}\prod_{j=1}^{j_{i}}\lambda_{ij}(X_{i})^{d_{ij}}(1-\lambda_{ij}(X_{i}))^{1-d_{ij}}  $$

which is equivalent to the likelihood of a binomial model with independent observations *d*_*ij*_, subject-specific probabilities *λ*_*ij*_(*X*_*i*_) for subject *i* experiencing the event in interval (*t*_*j*−1_,*t*_*j*_], and time-fixed covariates *X*_*i*_. Note that we do not make the assumption that the event indicators within a subject are independent and have a binomial distribution. Instead, we observe that the likelihood function for the discrete-time survival model under non-informative censoring can be represented using a binomial likelihood that assumes independent event indicators [27].

**Fig. 1:**
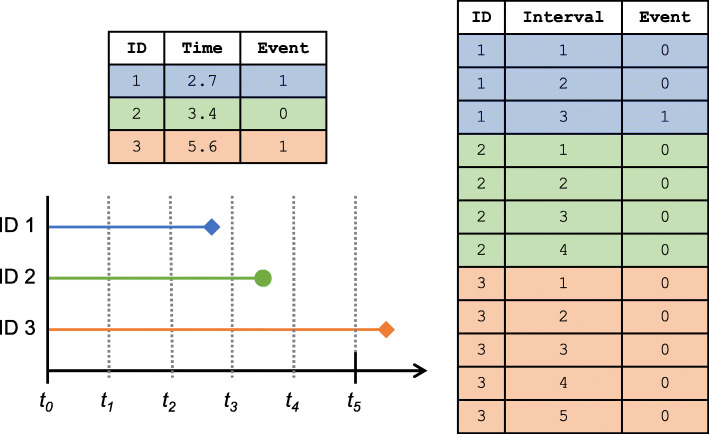
Example of a person-period data set (on right) created from continuous-time survival data (on left). In the timeline plot, circles indicate censoring and diamonds indicate events. The horizon of interest is *w*=5 and there are *J*=5 specified intervals defined as 1: *A*_1_=(*t*_0_,*t*_1_], 2: *A*_2_=(*t*_1_,*t*_2_], 3: *A*_3_=(*t*_2_,*t*_3_], 4: *A*_4_=(*t*_3_,*t*_4_], 5: *A*_5_=(*t*_4_,*t*_5_], whose endpoints are given by *t*_0_=0, *t*_1_=1, *t*_2_=2, *t*_3_=3, *t*_4_=4, *t*_5_=5. ID 1 experiences an event in interval 3 and thus in the person-period data set they have rows corresponding to the first three intervals and for the 3rd interval, their event status is a 1. ID 2 is censored in interval 4 and in the person-period data set they have row corresponding to the first 4 intervals and have an event status of 0 for all of them. ID 3 experiences the event at a time beyond the prediction horizon of interest, thus we administratively censor them at the prediction horizon and they have a row in the person-period for all intervals and have an event status of 0 for all of them

To construct this likelihood from our data, we need to convert it into a person-period data set, as depicted in Fig. 1. Subjects contribute a row for each time interval at which they are still at risk at the start, i.e., all *j* such that *T*>*t*_*j*−1_. Each record contains the subject’s failure indicator for experiencing the event during that interval (i.e., their event history indicator *d*_*ij*_), a copy of their baseline covariate vector *X*_*i*_, and a factor variable identifying the interval *A*_*j*_ to which the record corresponds.

Due to the binomial structure of the likelihood function in Eq. (2) the discrete survival time formulation is general and any algorithm that can optimize a binomial log-likelihood can be used to obtain parameter estimates. Thus, within this approach we can apply any method for computing the probability of a binary event and can choose from various binary classification methods, from traditional regression methods to more complex machine learning approaches. Estimation can be conducted by applying these methods to the described person-period data set. The resulting estimates can then be used in Eq. (1) to compute the predicted survival probability for a particular follow-up time.

The advantage of a discrete-time survival approach is that it does not require a proportional hazards assumption for the survival time distribution. As well, it provides a more intuitive interpretation since the hazard function represents the probability of experiencing the event in an interval given the person is alive at the start of the interval. Discrete-time models are also able to handle tied failure times without adjustments [26], as is required in Cox PH modeling due to its assumption of a continuous hazard in which ties are not possible [3].

### Parametric classification models

Cox (1972) proposed that since in discrete time the hazards, *λ*_*ij*_, are probabilities, they can be parameterized to have a logistic dependence with the predictors and time intervals [4, 27]. That is, we assume the predictors are linearly associated with the logistic transformation of the hazard (logit-hazard) instead of with the hazard probabilities themselves. Specifically, the conditional odds of experiencing failure in each time interval (*t*_*j*−1_,*t*_*j*_] (given that it has not yet occurred) is assumed to be linear function of the predictor and interval effects. This model is referred to as the *continuation ratio model*, and is specified as

3 $$  \log\left(\frac{\lambda_{ij}}{1-\lambda_{ij}}|X_{i}\right) = \alpha_{j} + \beta X_{i}  $$

where *α*_*j*_ is the logit of the baseline hazard for interval (*t*_*j*−1_,*t*_*j*_], and *β* describes the effect of the other covariates on the baseline hazard on the logit scale, as in a logistic regression. That is, for a binary predictor *X*_*ip*_, a positive (negative) coefficient estimate for *β*_*p*_ indicates an upward (downard) shift in the logit-hazard for those with *X*_*ip*_=1 from the logit-hazard for those with *X*_*ip*_=0. Thus, by taking the exponential on both sides of Eq. (3), we see that the odds is proportional for those with *X*_*ip*_=1 vs. 0. This property restricts the discrete hazards of both groups from crossing. This rules out the scenario in which predictors have protective short term effects on survival but are inferior at later time points. Notice also that this effect is not a function of time, and thus is assumed to be constant for all periods.

In the likelihood given in Eq. (2), the continuation ratio model assumes that the classification model for *λ*_*ij*_(*X*_*i*_) is a logistic regression. Estimation is conducted by obtaining maximum likelihood estimates of *α*_*j*_ and *β* using standard logistic regression software applied to the person-period data set. With estimates for *α*_*j*_ and *β*, we can then compute *λ*_*kj*_(*X*_*k*_) for a new subject *k* from the same population for all time intervals *j*, and then compute the survival prediction given in Eq. (1).

With the logistic model, when the magnitude of the hazards is small we find that the odds of failure approximate the probability of failure (i.e., *λ*_*ij*_/(1−*λ*_*ij*_)≈*λ*_*ij*_) and that hazards from the proportional odds model and proportional hazards model approximate each other. The continuation ratio model is shown to converge to the Cox model as the length of the discrete-time intervals goes to zero (i.e., the number of time intervals in a fixed period increases) [45].

In the parameterization *g*(*λ*_*ij*_|*X*_*i*_)=*α*_*j*_+*βX*_*i*_, we can also consider models with alternative link functions for *g* that are commonly used for binary outcomes. The Gompertz or grouped proportional hazards model that uses a complementary log-log link, log(− log(*λ*_*ij*_|*X*_*i*_)), is a discrete-time equivalent to a Cox PH model. Other parameterizations include the probit model (probit link), *Φ*^−1^(*λ*_*ij*_|*X*_*i*_), the Gumbel model (log-log link), − log(− log(*λ*_*ij*_|*X*_*i*_)), or the Exponential model (log link), log(*λ*_*ij*_|*X*_*i*_). For prediction purposes, the choice of link can be based on the predictive performance of the different models. It has been shown that the differences between the different models are small if the length of the discrete-time intervals are very short [45].

With both the continuous-time proportional hazards models and the parametric discrete-time models we make a proportionality assumption, which can be restrictive. Additionally, these models assume that the effect of the predictors on the transformed hazard is linear. An extension to allow for nonlinear effects is to use a semiparametric regression where the baseline hazard and covariate effects are specified as smooth, possibly non-linear functions of time, i.e., $\lambda _{ij}=f_{0}(j)+\sum _{p=1}^{P}f_{p}(X_{ip})$, and *f*_0_ and *f*_*p*_ can be chosen to be spline functions [46]. This can also be further extended to allow for non-proportional hazards and time-varying covariate effects by specifying *f*_*p*_ as a function of time, such as *f*_*p*_(*X*_*ip*_,*t*)=*f*_*p*_(*X*_*ip*_)·*t*. While these extensions can increase the flexibility of parametric modeling approaches, they may still not adequately capture the relationships that exist in the data. These relationships must be known and specified in the model-building process. Thus, there may be interest in exploring flexible, non-parametric approaches for describing the dependence between the hazard and predictors in the discrete-time survival setting.

### Machine learning classification models

An alternative approach for specifying this dependence is to use a non-parametric machine learning approach, such as random forests and neural network, with the form

$$ \lambda_{ij}(X_{i})=f(X_{i}, A_{j}) $$

where *f* is a particular machine learning algorithm or model, *X*_*i*_ is the subject-specific covariates, and *A*_*j*_ is the categorical variable indicating the time interval.

We fit a machine learning algorithm, *f*, to the person-period data set for the binary outcome of failure with the vector of predictors being the patient-specific covariates and the categorical variable identifying the discrete-time interval. For a new patient *k* with covariates *X*_*k*_, we can then obtain model-free estimates of the conditional probabilities $\hat {\lambda }_{kj}(X_{k})$ for each interval *j*=0,…,*J*. These estimates can then be plugged into Eq. (1), to get the survival prediction for patient *k* up to time *t*, i.e., *π*_*k*_(*t*).

The development of prediction models using a discrete-time framework has been demonstrated with semi-parametric methods [46], tree-based methods [29, 30], and neural networks [31, 33, 47]. With a discrete-time survival approach we are able to take advantage of the available software and computational efficiency for binary classification algorithms to predict the survival probabilities of interest. Within this class of more flexible prediction models, we can also consider penalized regression approaches such as lasso, ridge, and elastic net [48]. These approaches and their corresponding software implementations can accommodate additional binary classification methods without requiring method-specific estimation or optimization for obtaining the predicted survival probabilities.

#### Hyperparameter tuning

With machine learning algorithms, there is usually need to specify a *hyperparameter*. These are parameters that cannot be estimated but instead are specified to control the model training process. For example, in the random forest there are hyperparameters corresponding to the number of decision trees in the forest and the number of predictors considered at each split. Higher values for each of these can increase the computational time required for training the random forest. In the LASSO, the hyperparameter is a regularization parameter that is added to the ordinary least squares regression, such that higher values penalize the algorithm for including too many non-zero coefficients, thus performing variable selection.

The optimal hyperparameter values are not theoretically determined or based on prior knowledge, but instead can be *tuned* using empirical results. That is, for a grid of possible hyperparameter values or combinations of values when there are multiple hyperparameters, we can assess the performance of an algorithm under each set of values and select the values that lead to the best predictive performance. To avoid overfitting, we can perform hyperparameter tuning using cross-validation. We then select the hyperparameter values as those that optimize the performance of the cross-validated performance metric. These hyperparameter values are then used to train the algorithm on the training data set to build the prediction model.

With the use of binary classification models in the discrete-time survival setting, in addition to the hyperparameters required by the binary classification method employed, we also can tune the number of time intervals used for discretizing. The performance accuracy of discrete-time models has been shown to vary based on the number of intervals used [49]. Thus, instead of specifying the number of intervals, we can also consider this to be a hyperparameter for which an appropriate grid of possible values is explored to balance computational complexity and maximizing predictive performance.

The choice for the performance metric used for tuning should be the same as the metric used for overall performance assessment. Otherwise, tuned models might not have superior performance when compared to other models built using the same algorithm, and thus may not give a fair comparison when identifying the best method across multiple classes of prediction models and algorithms. We describe performance metrics for survival models in a later section.

### Alternative approaches

**Fig. 2:**
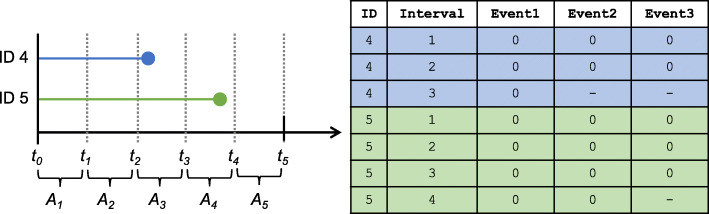
Demonstration of different specifications of censoring to create a person-period data set (on right) from continuous-time survival data of two censored individuals (on left). The horizon of interest is *w*=5 and there are *J*=5 intervals defined as *A*_*j*_=(*t*_*j*−1_,*t*_*j*_] for *j*∈{1,2,3,4,5}. Three different specifications are given for whether an individual contributes to a particular interval (Event1: intervals during which the individual is observed, Event2: intervals for which they survive at least half of the interval, Event3: intervals for which they survive the entire interval). ID 4 is censored in the first half of interval 3, so in the person-period data set they do not contribute to interval 3 in the Event2 and Event3 specification. ID 5 is censored in second half of interval 4, so in the person-period data set for interval 4 they contribute under Event2 specification but not under Event3 specification since they do not survive to the end of the interval

In an alternative approach to estimation, some recommend a different way of specifying the contribution of the censored subjects. If the censored individual’s survival time is in the second half of interval *A*_*j*−1_ or the first half of interval *A*_*j*_, i.e., $\frac {1}{2}(t_{j-2}+t_{j-1})\leq T_{i} < \frac {1}{2}(t_{j-1}+t_{j})$, we can consider their likelihood contribution as the product of the conditional survival probabilities from *j*=1,…,*j*−1 [16, 32]. Thus, a subject only gets counted for surviving an interval if they survive at least half that interval. This is in contrast with our current specification of including any intervals during which the censored subject has information observed. In the event that there is a lot of censoring present in the data or that there are fewer intervals specified with longer lengths, our current specification can bias the estimated hazard downward and the predicted survival towards one, making this alternative method a useful strategy to pursue. Another specification would be to consider observations that are censored in interval *A*_*j*_ as being censored at the end of interval *A*_*j*−1_. This mimics typical discrete-time data collection, where if an individual is lost to follow-up between measurement times *t*_*j*−1_ and *t*_*j*_, we would have last observed them at *t*_*j*−1_. We demonstrate how the person-period data set would be created under each of these specifications in Fig. 2.

There are also alternative approaches for prediction that exist within the discrete-time framework. In Eq. (1), we demonstrate the survival function can be written as a sequence of predictions from binary classification models. So far, the binary classification approach we have discussed models the conditional probabilities (or hazards) directly and uses the resulting estimates to compute the survival probability. Due to the dependence of the outcomes of these classification models (i.e., a death in a particular interval implies a death in all subsequent intervals), we can instead think of modeling the survival distribution directly as a series of dependent classification models. This can be considered a multi-task learning problem, where the related classification models are solved in parallel [50].

Yu et al. (2011) [51] proposed using multi-task logistic regression that specifies a logistic regression model for the conditional survival probability of each discrete-time interval and directly models the survival function by combining these local classification models. This approach estimates model parameters across these logistic regression models jointly using an optimization algorithm that uses two regularizers, a Euclidean norm regularization of the parameters to prevent overfitting, and a second regularizer that ensures the parameters vary smoothly over consecutive time intervals. Li et al. (2016) [52] also proposes a multi-task learning approach with logistic regression that uses an *ℓ*_2,1_-norm penalty to achieve a shared representation across the dependent classification problems that encourages sparsity and limits overfitting, and thus reduce the prediction error of each task. These methods have been shown to have superior performance compared to the Cox PH model [49, 51]. Other approaches have used a multi-task framework for discrete-time survival prediction by applying a deep learning approach to the series of binary classification tasks [47], and have been extended to accommodate competing risks [53].

Bender et al. (2020) [54] described using a Poisson regression framework applied to continuous survival data that specifies the hazard as a piecewise exponential PH model and uses a Poisson log-likelihood for estimation. One can then apply the range of machine learning algorithms that can optimize a Poisson (instead of a binomial) log-likelihood to perform estimation, which similarly to the described classification approach allows for a variety of existing algorithms to be applied to predicting survival. When continuous time is observed and we are willing to consider it in a discrete-time framework, this approach may be preferable due to its robustness to the choice of intervals. As well, we can use information about a subject’s partial observation in an interval instead of assuming that a censored subject survives the entirety of the last interval in which they were observed.

### Building discrete-time survival prediction models

**Fig. 3:**
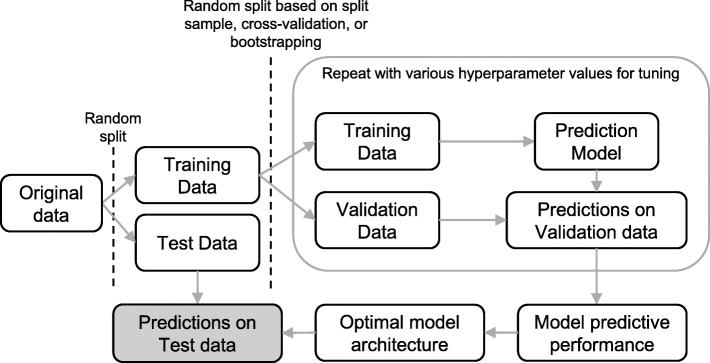
Visualization of the model building and testing process

A visualization of the model building and testing procedure for developing a survival prediction model is demonstrated in Fig. 3, and is comprised of the following components.

#### Data preparation

The data set should consist of individual-level records, such that there is a single row for each individual with a separate column for each baseline predictor. It must also contain a column of the subject’s follow-up time and a column indicating their event status at that time. If there are any subjects that are missing these two variables, they must be excluded from the data set. If there are any subjects that are missing other covariates, they can be removed from the data set to perform a complete case analysis. Alternatively imputation methods, such as using random forests [55, 56], can be applied to a covariate data set that excludes the follow-up time and event indicator columns to fill in missing predictor values.

#### Model training

To train our model we define an appropriate training data set based on our validation procedure. If we are using a 60/20/20 training/validation/test split we randomly select 60% of our full data set as the training data, 20% as a validation data set used to evaluate the model and select the hyperparameters, and the remaining 20% as a hold-out data set used for final model evaluation. If we are using a cross-validation or bootstrapping approach, then we nest the rest of the following components within a loop which randomly splits the data into folds (cross-validation) or resamples with replacement (bootstrapping), identifying the appropriate training and test data set for each iteration of the loop.

For the discrete-time survival models, we create the person-period data set for training the models using the training data. We discretize the continuous survival times into intervals. The intervals correspond to time points during follow-up at which the estimated hazard (conditional risk probability) is allowed to change. These intervals could be chosen based on the clinical context (i.e., times at which the conditional risk is expected to change). For example, in a clinical setting where patients are at high risk during a particular follow-up period and receive treatment and additional monitoring, one could increase the number of intervals during this period to reflect the frequently changing conditional risk. The horizon can be selected as the maximum of the prediction times of interest, or a clinically relevant follow-up time. Administrative censoring is then applied at this time horizon, such that individuals who experience an event or are censored after the horizon have a survival time that corresponds to the time horizon and are assumed to be event-free at that time. This limits the size of the resulting person-period data set and decreases the computational complexity. The resulting time range from baseline to the horizon is then divided into the specified number of intervals. The time intervals can be uniformly spaced on the range of event times within the horizon, but they do not have be equal in length and can be identified based on the quantiles of the event times. In this manuscript, we consider a general approach that requires specifying the number of intervals and then identifying the specific intervals based on the quantiles of the event times within a particular time horizon.

#### Hyperparameter tuning

Some machine learning methods require the specification of hyperparameters that must be tuned to optimize performance. In Additional File Table A1, we describe the hyperparameters for the methods considered in this manuscript. For the discrete-time prediction models, we additionally treat the number of intervals as a hyperparameter [49]. Tuning can be performed by identifying a reasonable range for the hyperparameter values, selecting a method by which to sample the values, and selecting a metric to assess performance. The model is fit for all of the sampled hyperparameter values and evaluated on the validation data. The tuned hyperparameter values are selected as those that optimize the performance metric. Methods of sampling the values include grid search, random search, and Bayesian optimization that uses the results from the previous iteration to improve the sampling of hyperparameter values for the current iteration [57–59]. The performance metric can be evaluated using an independent validation data set, cross-validation or bootstrapping.

#### Model testing

We apply the trained models to the test data set to obtain the predicted survival probabilities for the specified prediction horizons of interest. For the discrete-time models, we must create a person-period data set for the test data. We compute performance metrics to compare these predictions to the observed outcomes in the data set. This stage provides an assessment of the generalizability of the trained model to a new data set, and can identify issues such as overfitting. For a cross-validation or bootstrapping approach, the performance metrics from the model testing are averaged across multiple iterations. The results from this stage are compared for the different models to identify the optimal model or model architecture from which to obtain predictions from a new individual from the same population.

## Evaluating predictive performance

To compare models and choose the best approach for developing a prediction model we compute several metrics of predictive performance. Prognostic models are often assessed on two domains: (i) discrimination, which is their ability to distinguish between those with high and low risk of experiencing the event, and (ii) calibration, which is the agreement between the estimated and observed incidence of the event [60]. We assume that a model was fit to a training data set and that prediction accuracy is evaluated on an independent validation data set drawn from the same population as the training data set.

### Validation

The purpose of using an independent data set for evaluation is to avoid an overly optimistic assessment of performance that can occur if the training and validation data sets overlap. If an independent validation data set is not available, then internal validation can be performed on the available data using bootstrapping or cross-validation. In bootstrapping, we can consider the 0.632+ approach [61]. For *B* bootstrap samples the training data set is taken to be a random sample with replacement from the available data. Performance is then evaluated on a validation data set comprised of those that were not selected in the training data set. Performance metrics computed for each of the bootstrap samples are then averaged to obtain a bootstrap estimate of the metric. In *K*-fold cross-validation [37], we split the available data into *K* equal size data sets and then use each data set as the validation data set on which we assess performance of a model built on the combined remaining *K*−1 data sets. The cross-validation estimate of performance accuracy is then the average across the performance in the *K* validation data sets. This procedure can be repeated to reduce dependence on a particular split of the data, and an averaged estimate across the repeats can be used to obtain a repeated cross-validation estimate of predictive accuracy.

### Performance metrics

When assessing the prognostic accuracy of a prediction model using baseline covariates, we compute the prediction at baseline (i.e., time 0) for an event that occurs in the future. Thus, subjects start event free at time 0 and their outcome status can change over time. As a result, the metrics considered for assessing predictive accuracy for time-to-event outcomes are time-dependent and can be computed for different prediction time horizons, *w*. In the absence of censoring, we can use empirical estimates to quantify these metrics. However, it is possible that a subject is lost to follow-up before time *w*, in which case we cannot know for sure whether they experienced the event before *w*. Thus, estimation of these time-dependent metrics must account for censoring. Here, we consider two common approaches to measure the predictive accuracy of survival prediction models, area under the ROC curve (AUC) and Brier score. We define time-dependent measures of these metrics and describe their estimation.

#### Area under the ROC curve

A popular measure of the predictive accuracy of a predictive model is the area under the ROC curve. AUC, also referred to as the c-statistic or concordance index, is a summary measure of the sensitivity and specificity over a range of thresholds. This metric assesses the discriminative ability of the model, i.e., to distinguish between individuals with high and low risk of experiencing the outcome in the future. This can be interpreted as the probability that a randomly selected subject with the event (case) has a higher predicted probability than a randomly selected subject without the event (control). It is scale-free measure, with 0.5 indicating discriminative ability similar to chance and 1 indicating perfect discrimination.

The definition of AUC has been extended to a time-dependent setting using time-varying definitions of the true positive rate and false positive rate to accommodate the change in outcome status for different prediction windows. There are multiple definitions of the time-dependent AUC that can be considered for assessing discrimination [62]. We use a cumulative sensitivity/dynamic specificity (C/D) definition, which distinguishes between subjects who fail prior to a particular prediction window *t* (i.e., cases are defined by *T*_*i*_≤*t*) and those that are still event-free at that time (i.e., controls are defined by *T*_*i*_>*t*) [62, 63]. For two independent subjects *i* and *j*, the time-dependent AUC is given by

4 $$ \text{AUC}(t)=\text{Pr}(p_{i}(t)>p_{j}(t)|T_{i}\leq t, T_{j}>t)   $$

where *p*_*i*_(*t*)=1−*π*_*i*_(*t*) is the subject-specific probability of experiencing the event by time *t*. This is the recommended measure of discrimination when there is a specific period of time for experiencing the event of interest (e.g., within the first *t*=5 years of baseline) [64]. Based on its clinical relevance, the reporting of AUC using this definition is encouraged in clinical applications [65, 66].

#### Brier score

We can also assess performance using the expected Brier score, also known as prediction error [67–69]. This metric provides an overall performance measure by simultaneously assessing the calibration and discrimination of the prediction model. Lower values of the BS indicate better predictive performance. The Brier score can be seen as a mean square error of prediction and is given by

5 $$ \text{BS}(t)=E[\left(D(t)-{p}(t)\right)^{2}]   $$

where *D*(*t*)=*I*(*T*≤*t*) is the event status and *p*(*t*) is the estimated subject-specific probability of experiencing the event by time *t*. The Brier score has been shown to take its minimum value when the true event probabilities are used as predictions, but its maximum value is dependent on the cumulative incidence of the event of interest by time *t*. Instead, we can use a standardized version that scales the Brier score by that from a null model, *BS*_0_(*t*), that assumes that all subjects have the same predicted probability of the event regardless of their subject-specific predictors (e.g., the Kaplan-Meier estimate). This results in an R-squared type measure that represents the explained residual variation and is defined by *R*^2^=1−*BS*(*t*)/*BS*_0_(*t*), where higher values indicate better predictive performance. If we are interested in assessing the predictive performance of a model at multiple time points (e.g., for all times 0≤*t*≤*t*^∗^), we can integrate the Brier score with respect to a weight function, such as *W*(*t*)=*t*/*t*^∗^, and present an integrated Brier score, $\text {IBS}=\int _{0}^{t^{*}}BS(s)dW(s)$ [68]. In which case, the IBS can be used in the definition of *R*^2^ instead.

#### Time-dependent estimators

Estimation of these time-dependent performance metrics must account for the unobserved true outcome status for some individuals during the study period due to censoring. To account for the loss of information due to censoring, we reweight the individual contributions using the Kaplan-Meier estimator of the survival function of the censoring time, *G*(*u*)=Pr(*C*>*u*). Those that experience the event by time *t* get weight $1/\hat {G}(\tilde {T}_{i})$, those that are censored by time *t* get weight 0, and those that have a follow-up time beyond *t* get weight $1/\hat {G}(t)$.

To estimate Eqs. (4) and (5), we then use inverse probability of censoring weighting estimates of the time-dependent AUC and BS [63, 68] that are given by

$$\begin{aligned} \hat{\text{AUC}}(t)&=\frac{\sum_{i=1}^{n}\sum_{j=1}^{n}I(p_{i}(t)>p_{j}(t))\tilde{D}_{i}(t)\left(1-\tilde{D}_{j}(t)\right)\hat{W}_{i}(t)\hat{W}_{j}(t)}{\sum_{i=1}^{n}\sum_{j=1}^{n}\tilde{D}_{i}(t)\left(1-\tilde{D}_{j}(t)\right)\hat{W}_{i}(t)\hat{W}_{j}(t)}\\ \hat{\text{BS}}(t)&=\frac{1}{n\hat{S}_{\tilde{T}}(t)}\sum_{i=1}^{n}\hat{W}_{i}(t)\left(\tilde{D}_{i}(t)-\lambda_{i}(t)\right)^{2} \end{aligned} $$

where $\tilde {D}_{i}(t)=I(t\leq \tilde {T}_{i};\delta _{i}=1)$, $\hat {S}_{\tilde {T}}(t)=(1/n)\sum _{i=1}^{n}$$I(\tilde {T}_{i}>t)$, and weights to account for censoring are given by

$$\hat{W}_{i}(t)=\frac{I(\tilde{T}_{i}>t)}{\hat{G}(t)}+\frac{I(t<\tilde{T}_{i})\delta_{i}}{\hat{G}(\tilde{T}_{i})}.$$

The advantage of these estimators is that they are model-free and make no assumption about the correctness of the specification of the models used for computing the predicted probabilities. Computation of these metrics can be conducted in R using the Score function in the ‘riskRegression’ package.

## Experiments

We present a comparison of the continuous-time models, Cox PH and RSF, and discrete-time survival prediction models built using the following classification algorithms: logistic regression, conditional inference random forest, gradient boosting machine, elastic net, support vector machine, and neural network.

We create the discrete-time intervals based on the quantiles of the event times in the data set that are within the maximum prediction horizon of interest. We additionally standardize the predictors by centering and scaling them. We perform hyperparameter tuning using a Bayesian optimization search scheme within a provided grid of acceptable values [59]. The hyperparameters were tuned over the validation data set to minimize the time-dependent Brier score, or the integrated Brier score when multiple time points were specified. Depending on the method for validation, predictive performance is demonstrated in the test data set, or averaged across cross-validation folds.

### Performance comparison

To compare the performance of these methods in various settings, we assess the predictive performance of the discrete-time and continuous-time methods in multiple publicly-available data sets. The data set characteristics are described in Table 2, and vary in terms of sample size, number of predictors, and censoring. We present 5-fold cross-validation performance metrics for predicting survival probability at the median survival time (Table 3, Additional File Table A2).

**Table 2:** Description of data sets used to assess external validity

Data set	Sample size	Predictors	Prop. Censoring	Median survival time
flchain	6,524	13	70%	12.7 years
metabric	1,904	9	42%	197 months
nwtco	4,028	5	86%	6.4 years
colon	888	11	52%	6.4 years
pbc	276	17	80%	6.7 years

metabric (Molecular Taxonomy of Breast Cancer International Consortium) is obtained from the Python DeepSurv package [42]. The remaining data sets are from the R survival package [22]: flchain (Assay Of Serum Free Light Chain), nwtco (National Wilm’s Tumor Study), colon (Chemotherapy for Stage B/C colon cancer), pbc (Mayo Clinic Primary Biliary Cholangitis Data)

**Table 3:** Cross-validated R-squared Brier score for continuous and discrete-time survival prediction models in multiple data sets

Method	metabric	flchain	nwtco	colon	pbc
Cox PH	0.140	0.351	0.112	0.111	0.334
RSF	0.133	0.344	0.140	**0.128**	0.389
Logistic regression	0.136	0.352	0.114	0.112	0.328
Elastic net	0.136	0.350	0.116	0.114	0.353
Support vector machine	0.070	0.101	0.018	0.046	0.349
GBM	0.128	0.364	0.149	0.121	0.378
Neural network	**0.163**	**0.366**	0.136	0.116	**0.415**
CForest	0.138	0.343	**0.147**	0.123	0.377

Higher values indicated better predictive performance. Bold values indicate method with best predictive performance (highest R-squared) in a particular data set. CForest: conditional inference random forest; GBM: gradient boosting machines; PH: proportional hazards; RSF: random survival forest

In terms of *R*^2^ based on the Brier score (Table 3), overall we find that both discrete- and continuous-time methods performed well across the different data sets, with the exception of the discrete-time support vector machine approach that performed much worse than the other methods in all but one data set. A discrete-time method had the best predictive performance in all but one of the data sets. The best discrete-time methods were those that use a neural network or conditional forest classification approach. The discrete-time approach using logistic regression performed similarly to the Cox proportional hazards model for all data sets. We find similar results for AUC (Additional File Table A2). The optimal number of intervals for a particular discrete-time model varied across data sets as well as for the different discrete-time models within a particular data set (Additional File Table A2).

### Demonstration of pipeline

We additionally demonstrate the modeling process and resulting predictions in the R programming environment using the data sets pbc and colon from the ‘survival’ package, with code available at: https://github.com/ksuresh17/autoSurv. The binary classification models are fit using the ‘caret’ package, allowing for easy extension to other classification methods as they become available. We use these two examples to demonstrate alternate parameterizations of censoring during model training, and different methods of evaluation (cross-validation, test/training split) for single or multiple prediction times of interest.

#### PBC data

The pbc data set consists of 276 patients with primary biliary cirrhosis who have complete information on 17 clinical and demographic predictors. The median follow-up time in this data set is 6.7 years and the censoring rate is 60%. Cross-validation is performed within the training data set to tune the hyperparameters, and predictive performance metrics are reported as the average across the cross-validation folds. The models were tuned for predicting survival probability at seven years using the Brier score. For the discrete-time models, the censored observations are treated as surviving an interval if they survive at least half of the interval.

The cross-validated performance metrics are presented in Table 4. We report the tuned number of intervals for the discrete survival models and find that these vary across the different models. In Additional File Fig. A1, we present the interval endpoints in the full data based on the optimal number of intervals for each discrete-time method. The cross-validated AUCs are similar, ranging from 0.83-0.88, indicating good discrimination for all models. In comparing the continuous-time models to the discrete-time models, we find that several discrete-time survival models outperform the continuous-time Cox PH model. The neural network applied in the discrete-time framework has the best overall performance with the highest *R*^2^, and the continuous-time random survival forest has the highest AUC.

**Table 4:** Cross-validated time-dependent R-squared measure of Brier score and AUC for the prediction models applied to the pbc test data to predict survival at *w*=7 years

Method	Type	No. Intervals	*R*^2^	AUC
Neural network	Continuous	15	**0.409**	0.859
RSF	Continuous	-	0.392	**0.880**
CForest	Discrete	8	0.377	0.869
GBM	Discrete	6	0.363	0.826
Support vector machine	Discrete	5	0.354	0.854
Elastic net	Discrete	25	0.345	0.845
Cox PH	Discrete	-	0.332	0.831
Logistic regression	Discrete	11	0.329	0.831

Higher values of AUC and *R*^2^ scores indicate better performance. Results are sorted by decreasing *R*^2^ (best to worst). Type indicates whether the method is applied to continuous- or discrete-time data. AUC: area under the ROC curve; CForest: conditional inference random forest; GBM: gradient boosting machines; PH: proportional hazards; RSF: random survival forest

**Fig. 4:**
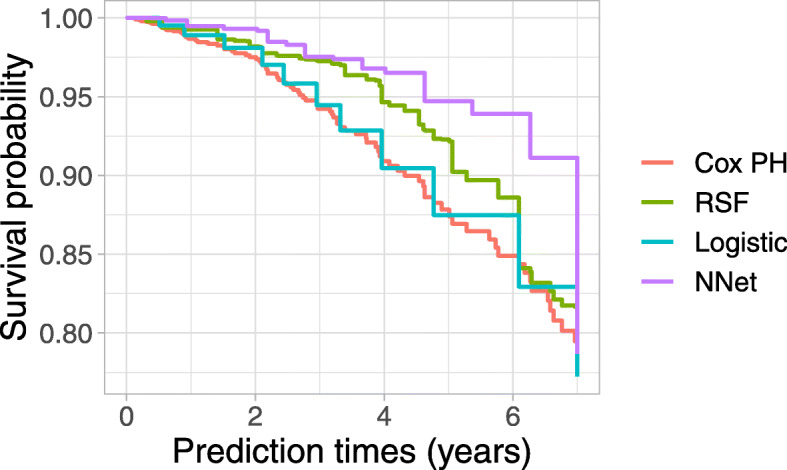
Predicted survival probabilities for a randomly selected individual in the pbc data set with continuous-time models, Cox PH and RSF, and discrete-time models using logistic regression and a neural network. NNet: neural network; PH: proportional hazards; RSF: random survival forest

We examine the predicted survival probabilities for an individual in Fig. 4 for the continuous-time models, Cox PH and RSF, and for the discrete-time models that use a logistic regression and neural network. We can see that the survival estimates from the discrete-time models are a step function with the number of steps being equivalent to the number of tuned intervals. The steps in the Cox PH model and RSF correspond to event times in the data set. These individual-specific predictions can be used in clinical practice to identify whether the patient is at increased risk of death during follow-up and to aid in making treatment or monitoring decisions at baseline.

#### Colon data

The colon data set has complete information for 11 baseline predictors for 888 patients with colon cancer that participated in a trial studying adjuvent chemotherapies. The median follow-up time in this data set was 6.4 years and the censoring rate 52%. We use this data set to demonstrate the comparison of model performance for predicting survival across multiple prediction time points (i.e., 1, 2, 3, 4, and 5 years). We use a 60/20/20 training/validation/test split sample. The models were tuned using the integrated Brier score over the prediction times of interest. The censored observations in the discrete-time models were considered to survive an interval if they were at risk at the start of the interval.

**Fig. 5:**
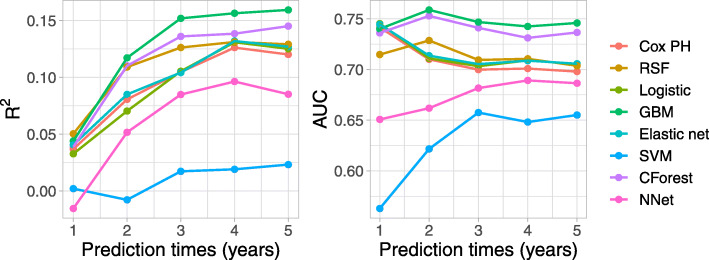
Test set time-dependent R-squared measure of Brier score (left) and AUC (right) for prediction models applied to the colon data set. Higher values indicate better predictive performance. The R-squared IBS over the 5 time points for the different models are in decreasing order: GBM (0.132), CForest (0.119), RSF (0.105), GBM (0.105), Elastic net (0.101), Cox (0.098), Logistic regression (0.096), Neural network (0.069), SVM (0.010). AUC: area under the ROC curve; CForest: Conditional inference random forest; GBM: gradient boosting machines; NNet: neural network; PH: proportional hazards; RSF: random survival forest

We present the time-dependent AUC and Brier score in Fig. 5. Applied to this data set, we find that several of the discrete time-to-event models perform better than the continuous-time models, Cox PH and RSF. The random forest in the discrete-time framework has better performance than the random survival forest. The Cox PH model has similar predictive performance to the logistic regression across the time points. The SVM discrete-time approach has poor performance based on both AUC and Brier score at all times.

## Discussion

Survival prediction models predict the risk of a future event and can be used in biomedical research to make treatment and monitoring decisions. These prediction models built using time-to-event data must account for censoring to avoid biased and inefficient predictions. Machine learning methods for classification have been adapted to accommodate censoring, but require the specification and optimization of a method-specific survival function. Thus, implementation of these methods is often found in different software packages. As well, some ML survival methods return the predicted survival time which can be inaccurate [68], or the risk score which requires estimation of the baseline hazard to be used for obtaining predicted survival probabilities. Here, we propose the use of discrete-time survival models to provide an alternate model architecture for predicting survival probabilities compared to the traditional continuous-time survival models.

An advantage of discrete-time prediction models over the commonly used Cox PH model is that they are able to handle ties in event times naturally. As well, any binary classification method can be implemented within the framework without requiring a model-derived specification of the survival function. This provides additional flexibility in being able to specify a method that does not require a proportional hazards assumption. Specifically, we consider nonparametric machine learning classification methods to take advantage of their ability to capture complex relationships between the predictors and the outcome, their lack of required prior specification of predictor relationships and behaviour, and their current availability in open-source software.

In an application to multiple publicly available data sets, we demonstrate that the discrete time-to-event methods have similar or sometimes superior performance to the Cox PH and random survival forest applied to continuous-time data. By treating as a tuning parameter the number of intervals used to create the person-period data set on which we train the discrete-time models, we are able to optimize performance for each applied classification approach. We can then at the very least achieve similar predictive performance between a logistic regression model and a Cox PH, which become approximately equivalent as the width of the discrete-time intervals decreases (i.e., the number of intervals increases). The optimal number of intervals varied both within a data set across the different discrete-time methods and for a particular method across the different data sets. The support vector machine method applied within the discrete-time framework had consistently poor performance in many data sets with the exception of the smallest data set. Support vector machines have been shown to have poor performance in settings with imbalanced data [70], and by using a person-period data set we are potentially creating greater imbalance as the number of intervals increases and the number of events per interval decreases. The relative performance of the ML classification methods varied based on the data application. Thus, care should be taken to appropriately train and assess the considered classification methods for each specific application (e.g., specifying reasonable ranges for the hyperparameter search space).

The disadvantage of the discrete-time approach is that compared to the Cox PH model we are unable to obtain an interpretation of the relationship between the predictors and the survival probabilities. This is a common phenomenon with machine learning methods applied to continuous survival data as well. Thus, if there is interest in both inference and prediction, model-agnostic explainer methods can be considered [71, 72]. Additionally, the discrete-time survival method increases computational burden. As the number of discrete-time intervals increases so does the size of the person-period data set that includes a row for each person for each interval at which they are still at risk within the prediction horizon of interest. Thus, in comparing the conditional forest implemented in the discrete-time framework to the random survival forest, the conditional forest is applied to the larger person-period data set and takes more time to compute the survival probabilities. In our applications to data, we limited the maximum number of intervals considered in the tuning process to 25 and were able to achieve comparable predictive performance to the continuous-time methods. By tuning the number of intervals and selecting the endpoints based on quantiles of event times, the resulting optimal intervals are driven by the particular data set and prediction method and may have limited clinical relevance.

While we have demonstrated that the discrete-time models had similar or superior predictive performance in our applications to Cox models and random survival forests, these results may not be generalizable to other data sets, settings, or continuous-time prediction methods. Here, we use these approaches to obtain predicted survival probabilities for time points during an individual’s follow-up. However, other prediction approaches may be more appropriate if the goal for clinical implementation is to predict a risk score (e.g., gradient boosted Cox) or survival time (e.g., parametric survival models), or examine variable importance (e.g., random survival forest). Thus, when developing a prediction model for time-to-event outcomes, in addition to assessing predictive performance, models should be evaluated based on their appropriateness to obtain the desired interpretation, visualization, and clinical implementation.

There are alternative approaches for obtaining survival predictions applied to a discrete-time framework. The multi-task learning approach creates a sequence of classification models, such as logistic regression or neural networks [51, 52], however requires a classification method-specific definition of the survival function for optimization. Thus, it lacks the scalability to accommodate multiple and newly developed ML classification methods that is possible with the approach considered in this manuscript. There are also various extensions of discrete-time survival models to accommodate other characteristics of time-to-event data, such as competing risks or time-dependent covariates [26, 73]. Extensions to using ensemble approaches for prediction within the discrete-time framework have also been explored [30, 74]. Thus, the flexibility and versatility of the discrete-time survival models make it a useful option to consider when developing a prediction model for survival outcomes.

## Conclusions

In this manuscript, we describe the advantages of using machine learning within a discrete-time framework for predicting survival probabilities. We demonstrate the use of these methods using publicly available data sets and compare their predictive performance to the continuous-time Cox PH model and random survival forest. The ability of the discrete-time methods to achieve similar or sometimes superior predictive performance when computing predictions at a single time or multiple time points of interest indicate that they should be considered when developing survival prediction models. By presenting software and a model development framework for discrete-time prediction models, we aim to encourage their inclusion in identifying a model architecture for optimally predicting survival in various time-to-event applications.

## Supplementary Information

**Additional file 1** Supplementary Material.

## Data Availability

In this manuscript we use publicly available data in the R programming environment [35]. The described software for implementation is available at https://github.com/ksuresh17/autoSurv. The referenced data sets are publicly available in the following software packages: R survival (flchain, nwtco, colon, pbc) [22], Python DeepSurv (metabric) accessible at https://github.com/jaredleekatzman/DeepSurv.

## Abbreviations

AFT: accelerated failure time
AUC: area under the ROC curve
BS: Brier score
CForest: conditional inference forest
GBM: gradient boosting machine
IBS: integrated Brier score
PH: proportional hazards
ML: machine learning
NNet: neural network
ROC: receiver operating characteristic
RSF: random survival forest
SVM: support vector machine
