# Supplementary material for: Survival prediction models: an introduction to discrete-time modeling
Source: BMC Med Res Methodol. 2022 Jul 26;22:207. doi: 10.1186/s12874-022-01679-6 (PMC9316420; doi:10.1186/s12874-022-01679-6)
Supplement: Supplementary file 1 — Additional file 1 Supplementary Material. [file 12874_2022_1679_MOESM1_ESM.pdf]

Survival prediction models: an introduction to discrete-time modeling
Supplementary Material

Krithika Suresh, Cameron Severn, Debashis Ghosh

Table A1: Hyperparameters for implemented machine learning algorithms and their range of values used in tuning.

Method	Hyperparameter	Values
Random survival forest	nodesize: average terminal node size	[1, 15]
	mtry: number of randomly selected predictors to try at each split	[2, # predictors]
Gradient boosting machines	n.trees: number of boosting iterations	[50, 500]
	interaction.depth: maximum tree depth	[1, 3]
	shrinkage: learning rate	[0.001, 0.1]
	n.minobsinnode: minimum size of terminal node	[1, 20]
Elastic Net	alpha: mixing percentage	[0,1]
	lambda: regularization parameter	[0,1]
Conditional inference forest	mtry: number of randomly selected predictors to try at each split	[2, # predictors]
Support vector machines	cost: misclassification cost	[0,30]
	sigma: smoothing parameter	[0.01,0.2]
Neural network	size: number of hidden units	[1, 10]
	decay: weight decay (regularization parameter)	[0.01, 0.5]

For all discrete-time survival models, the number of intervals was tuned over the range [5, 25].

Table A2: Cross-validated AUC and optimal number of intervals for continuous and discrete-time survival prediction models in multiple data sets.

	metabric		flchain		nwtco		colon		pbc	
Method	AUC	Intervals	AUC	Intervals	AUC	Intervals	AUC	Intervals	AUC	Intervals
Cox PH	0.723	-	0.836	-	0.708	-	0.698	-	0.831	-
RSF	0.718	-	0.832	-	0.717	-	0.710	-	**0.880**	-
Logistic regression	0.718	22	0.837	6	0.713	6	0.699	7	0.831	11
Elastic net	0.718	25	0.836	5	0.715	5	0.702	5	0.845	25
SVM	0.671	7	0.731	6	0.473	5	0.635	5	0.854	5
GBM	0.714	17	**0.844**	24	0.731	6	**0.718**	5	0.826	6
Neural network	**0.738**	15	0.842	6	0.737	13	0.708	22	0.859	15
Cforest	0.721	5	0.833	7	**0.738**	7	0.709	5	0.869	8

Higher values indicated better predictive performance. Bold values indicate method with best predictive performance (highest AUC) in a particular data set. Cforest: conditional inference random forest; GBM: gradient boosting machines; PH: proportional hazards; RSF: random survival forest; SVM: support vector machine

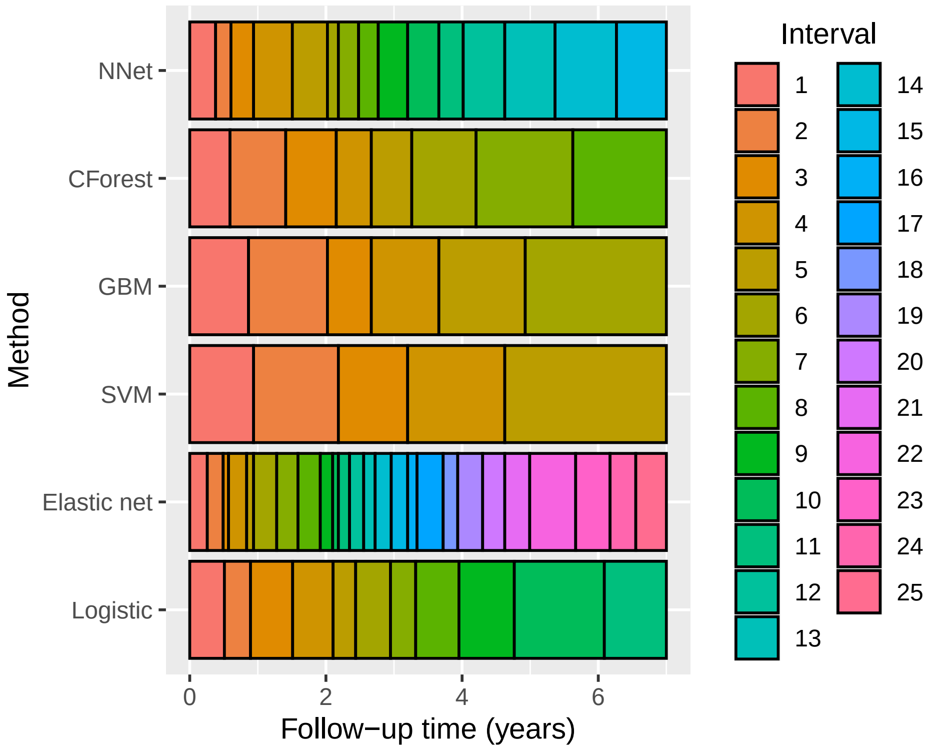

Figure A1: Interval endpoints in the pbc data set for the optimal number of intervals for each prediction method. Endpoints are determined based on the quantiles of event times within the time horizon of w=7 years.
